# The reproductive biology of *Ellochelon vaigiensis* from the Vietnamese Mekong Delta

**DOI:** 10.1002/ece3.11033

**Published:** 2024-02-14

**Authors:** Quang Minh Dinh, Ngon Trong Truong, Ton Huu Duc Nguyen, Tran Thi Huyen Lam, Tien Thi Kieu Nguyen, Zeehan Jaafar

**Affiliations:** ^1^ Department of Biology, School of Education Can Tho University Can Tho Vietnam; ^2^ Department of Molecular Biotechnology Institute of Food and Biotechnology, Can Tho University Can Tho Vietnam; ^3^ Institute of High Quality Biotechnology Food Technology Cuu Long University Vinh Long Vietnam; ^4^ Department of Biology An Khanh High School, An Khanh Ward Can Tho Vietnam; ^5^ Department of Biological Sciences National University of Singapore Singapore Singapore

**Keywords:** breeding season, fecundity, length at 50% maturity, squaretail mullet, synchronous spawner

## Abstract

The Squaretail mullet, *Ellochelon vaigiensis*, is a commercial fish species distributed throughout the Indo‐Pacific region. This species tolerates wide variations in salinity, having been reported from both freshwater and marine habitats. Although economically significant, there is little information on its reproduction, especially in the Vietnamese Mekong Delta (VMD), where it is heavily extracted. Our study elucidates the breeding pattern, seasonality, first length at maturity, as well as potential and relative fecundity of this species. Fish specimens were collected by trawl nets from November 2020 to October 2021 at four estuarine sites within the VMD. We found this species to be a synchronous spawner, with peaks in reproductive activity from June to September. In the populations we surveyed, there was significant dominance of males (1.26:1.00) based on 942 fish samples (526 males and 416 females). The length at 50% maturity for females was significantly higher than for males at all sites and ranged from 10.6 to 19.3 cm. The diameter of the eggs examined for this species exhibited slight variation, from 0.43 to 0.54 mm. The potential fecundity ranged from 246,254 ± 35,878 to 411,970 ± 54,370 eggs, corresponding to female length and weight of 26.7–32.6 cm and 102.97–234.19 g, respectively. Relative fecundity values were highest at Thanh Phu, Ben Tre (6388 ± 605 eggs), and Dong Hai, Bac Lieu (6461 ± 637 eggs), followed by Tran De, Soc Trang (4729 ± 408), and were found to be lowest at Duyen Hai, Tra Vinh (3117 ± 223 eggs). Information on reproductive behavior in this species has far‐reaching impacts on sustainable extractions, stock conservation, and aquaculture.

## Graphical abstract

*Ellochelon vaigiensis* is a synchronous spawner, with peaks in reproductive activity from June to October. The length at 50% maturity for females is significantly higher than that for males at all sites and ranges from 10.6 to 19.3 cm. The diameter of the eggs examined for this species exhibited slight variation, from 0.43 to 0.54 mm. The potential fecundity ranged from 246,254 ± 35,878 to 411,970 ± 54,370 eggs, corresponding to female length and weight of 26.7–32.6 cm and 102.97–234.19 g, respectively. Relative fecundity values peaked at Thanh Phu, Ben Tre (6388 ± 605 eggs) and Dong Hai, Bac Lieu (6461 ± 637 eggs), followed by Tran De, Soc Trang (4729 ± 408), and were found to be lowest at Duyen Hai, Tra Vinh (3117 ± 223 eggs).

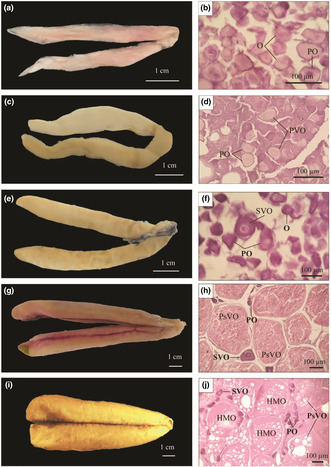

## 1 INTRODUCTION

The teleost family Mugilidae, commonly known as “mullets,” comprises 20 genera and 75 species (Nelson et al., 2016) that are ecologically and economically important. Mullets are distributed in temperate and tropical regions and occur in freshwater and marine habitats (Nelson et al., 2016). They are significant contributors to the energy flow of food webs in estuarine ecosystems (Rahman et al., 2016); gray mullets (*Mugil cephalus*) for example, are bottom‐dwelling species that consume decomposed organic matter, algae, and diatoms (El‐Marakby et al., 2006; Mondal et al., 2015). In 2016, the global yield of mullets totaled 763,291 tons, of which 76.8% were from capture fisheries (585,959 tons) and about 23.2% from aquaculture (177,332 tons) (FAO, 2018).

The commercial value of mullets varies by country. In Tunisia, Egypt, and Taiwan, they are sought‐after and demand high prices, but the opposite is true in Spain, France, and Australia (Whitfield et al., 2012). Still, mullets are essential dietary components that contribute significantly to the protein intake of coastal and marine communities. Mullets are considered oily fishes, with relatively few bones (Brian, 2017), but the greater economic worth of mullets are as bait fisheries and the roe‐carrying females (Ben Khemis et al., 2019). Salted and dried roe has become a highly valued product with growing global demand (Bledsoe et al., 2003). Mullet flesh confers an excellent source of nutrition for humans; specifically, 150 g can meet most of the weekly requirements for essential fatty acids, such as EPA þ DHA (Ben Khemis et al., 2019).

In Vietnam, 22 species of mullets, in five genera, inhabit coastal waters, estuaries, and brackish lagoons. Of these, seven species, including *Ellochelon vaigiensis*, are considered economically important as popular food fishes (Tran et al., 2015), but their reproductive behavior is poorly understood. Mullets have been reported to grow poorly in freshwater but well in brackish water or water bodies with higher salinity (Cardona, 2016). They are most affected by salinity shock at low temperatures (Cardona, 2016). In contrast, fingerlings are usually concentrated in freshwater or pale brackish environments in subtropical areas all year round (Cardona, 2016). Mullets that are mature undergo reproductive migration, and habitat shifts occur seasonally. During the spawning season, mullets migrate to the sea to avoid freshwater areas.

**FIGURE 1:**
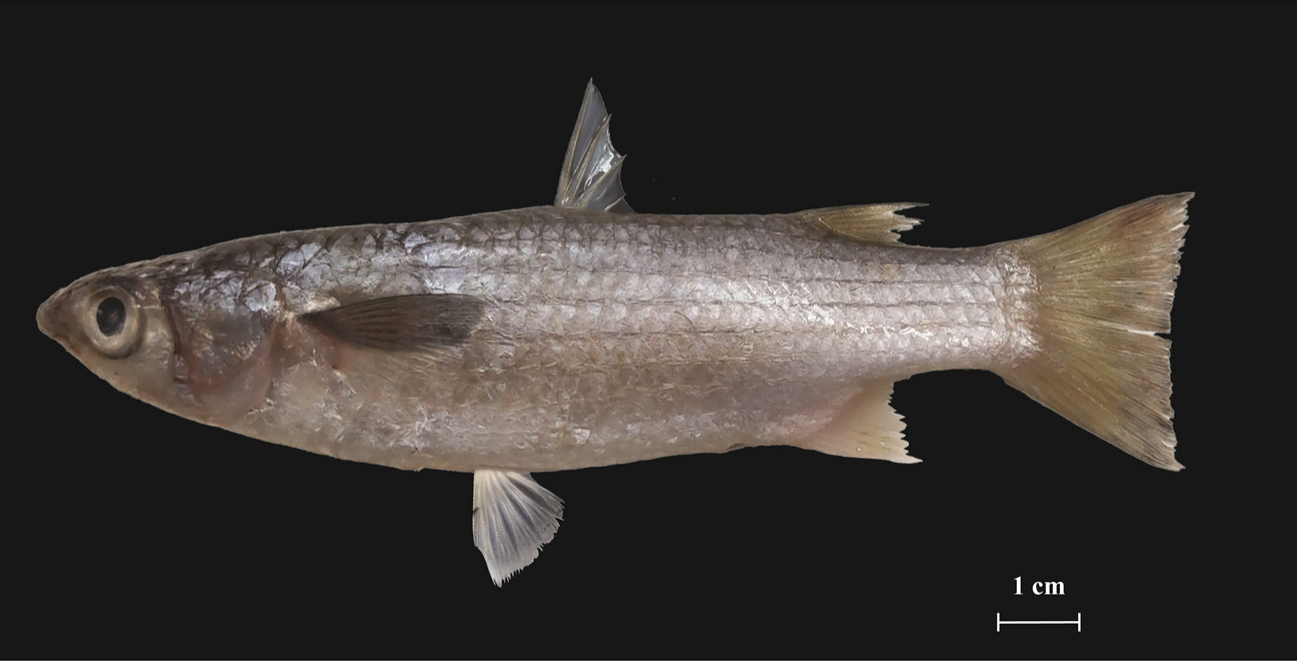
The photo of *Ellochelon vaigiensis*.

The Squaretail mullet, *Ellochelon vaigiensis* (Quoy and Gaimard, 1825) (Figure 1), is considered economically vital as it is frequently consumed throughout its range. Wijeyaratne and Costa (1990) suggested that *E. vaigiensis* is a suitable candidate for brackish aquaculture because its growth rate, maximum length, and condition factor are high and ideal for aquaculture. In addition, the critical thermal maximum value of squaretail mullet approximates some of the most thermally tolerant vertebrates at 43.8–44.8°C and can, therefore, survive habitats with extremely high temperatures (Bennett & Beitinger, 1997; Eme et al., 2011).

Some studies on *E. vaigiensis* in the Mekong Delta have been published. The growth pattern of *E. vaigiensis* is negative allometry because the *b* value is <3 (2.13–2.68), and this fish is well adapted to the environment as the CF value approaches the well‐being threshold of 1 (0.97–1.13) (Dinh, Truong, Nguyen, Tran, et al., 2022). This squaretail mullet is an algi‐omnivorous with the food composition of mainly Bacillariophyta, followed by detritus (30.3%) and Cyanophyta (18.3%) (Dinh, Truong, Nguyen, Lam, et al., 2022). Nguyen et al. (2022) revealed that *E. vaigiensis* has a small mouth, thick gill rakers, and a short belly but a long gut, indicating a predominantly herbivorous diet. The salinity variation between two ecological regions may affect the biological parameters of *E. vaigiensis* populations. Namely, the growth index of this species in STBL (2.74) is higher than in BTTV (2.72), while the life expectancy in BTTV (6.52 years) is higher than in STBL (5.36 years). The BTTV and STBL populations of this mullet in the Mekong Delta currently have not been subjected to overfishing (Dinh et al., 2023).

Despite its economic and ecological importance, little is known about the reproduction of *E. vaigiensis*: In what season does it reproduce? How does it reproduce? What is its fertility rate? The stocking of cultured mullets is primarily based on fry harvested from the wild (Ben Khemis et al., 2019). A study on the population from Northern Queensland, Australia, reveals this species spawns from February to March with batch fecundity of 0.805–1.204 × l0^6^ and oocyte diameter of 0.54 ± 0.05 standard error (SE) mm (Grant & Spain, 1975) and the spawning season lasting from May to February in India (Chidambaram & Kuriyan, 1952). However, information essential to the management of wild and captive fisheries, such as length at 50% maturity (*L*_m_), sizes of spawning individuals, the sex ratio in the populations, and oocyte and spermatocyte developments, are unavailable (Frank & Leggett, 1994; Morita & Morita, 2002). In addition to determine the extraction limits to ensure sustainability, such data can inform regulation of catch sizes of fish and the mesh sizes of nets used in their capture (Roff, 1981). This study represents an essential reference on the reproduction biology of *E. vaigiensis* in the Vietnamese Mekong Delta (VMD) to achieve the sustainable exploitation of this fishery resource.

## 2 MATERIALS AND METHODS

### 2.1 Study sites

**FIGURE 2:**
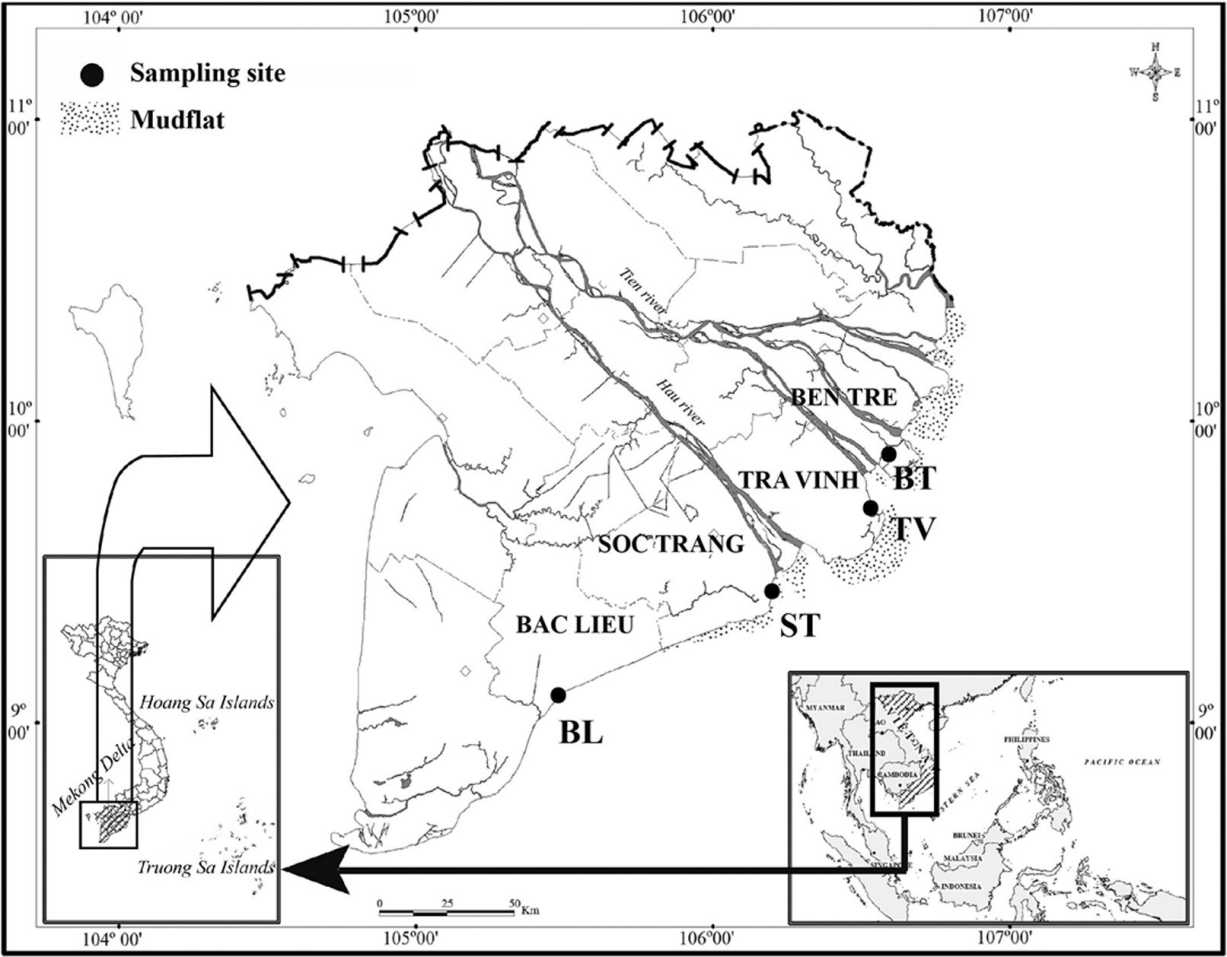
The sampling sites in the Vietnamese Mekong Delta (•: Sampling area; BT: Thanh Phu, Ben Tre; TV: Duyen Hai, Tra Vinh; ST: Tran De, Soc Trang; BL: Dong Hai, Bac Lieu; modified from Dinh (2018)).

Specimens of *E. vaigiensis* were sampled at four mangrove sites along the Hau river monthly between November 2020 and October 2021: Thanh Phu, Ben Tre (BT, 9°57′01.3″N 106°31′43.1″ E); Duyen Hai, Tra Vinh (TV, 9°40′29.5″ N 106°34′49.5″ E); Tran De, Soc Trang (ST, 9°26′19.7″ N 105°10′48.1″ E); and Dong Hai, Bac Lieu (BL, 9°05′50.5″N 105°29′54.7″ E) (Figure 2). With low‐lying terrain, the Mekong Delta receives nutrient‐rich silt from the Mekong and Dong Nai rivers (Veettil et al., 2019), and has the largest mangrove area in Vietnam with a total of 69 species (Hong & San, 1993). Although the area of mangroves is small compared to many other forest ecosystems, the mangrove ecosystem is essential to maintaining ecosystem services of coastal habitats. Mangrove forests in the Mekong Delta are distributed along the coasts of all coastal provinces, with the largest concentrated area in Ca Mau Province and narrow coastal strips in other provinces, ranging from a few meters to about 1 km adjacent to the sea or along rivers and canals (Tinh et al., 2022). These estuarine mangrove areas are characterized by several dominant flora – *Avicennia marina*, *Bruguiera gymnorrhiza*, and *Sonneratia caseolaris* (Nguyen et al., 2020). The area experiences a semi‐diurnal tidal cycle and an average daily temperature of about 28.5°C (25.6–30.6°C). The wet season occurs between June and December; the rain accumulated during this period accounts for 90% of the total annual rainfall. The dry season is from January to May, and the sparse showers account for the remaining 10% of annual rainfall (Le et al., 2006). The pH values of the water along the lower Hau River of the study sites were recorded in the range of 7.5–7.6 and did not differ between sites. Conversely, the salinity differed between sites, reported between 8.2‰ and 16.7‰, highest in BL (16.7‰), followed by ST (15.2‰), TV (10.4‰), and lowest in BT (8.2‰).

### 2.2 Capture and examination of fish specimens

Mullets were sampled during the final 3 days of each month. Trawl nets were set at high tide and retrieved 2 h later. All fishes were euthanized with MS222 and stored in 5% buffered formalin before they were transported to the laboratory. The study was approved by the Scientific Committee of the School of Education, Can Tho University (No. BQ2021‐05/KSP) after animal welfare assessments.

At the laboratory, the total length (TL, cm) and weight (*W*, g) of each specimen were recorded. Fishes were then eviscerated, and the gender was determined based on internal and external gonad morphology. The ovaries and testes were weighed to the nearest 0.01 mg; the stages of gonad development followed Dinh et al. (2020). A total of 25 ovarian and 25 testicular samples (five samples for each stage) representing the stages of gonadal development from I‐V were stained (see Carleton et al., 1980; Dinh & Nguyen, 2022) for histological observations. Description of developed oocytes and spermatocytes follow Yamamoto (1956) and Yamazaki (1965).

The gonadosomatic index (GSI) was obtained from the equation GSI = 100 × (*G*/*W*) (*G*: gonad weight, *W*: fish body weight) (Sturm, 1978). The breeding season of mullet was inferred by analyzing the GSI value and the frequency of occurrence of mature gonads (Alonso‐Fernández et al., 2011; Dinh & Le, 2017).

The first lengths at maturity (*L*_m_) of both males and females were estimated from the expression: *P* = 1/(1 + exp[−r × (TL−*L*_m_)]) (*P*: proportion of mature individuals in a length class; TL: fish total length; and *r*: model parameter) (Zar, 1999).

The gravimetric method was used to estimate the potential fecundity – the number of oocytes released by the female during the breeding season (Hunter et al., 1985). The formula PF = (*n × G*)*/g* (*n*: number of oocytes in sub‐sample; *g*: weight of sub‐sample; and *G*: ovarian weight) (Bagenal, 1967) was used to estimate the batch fecundity from 20 mature ovaries for each sampling site. Three 1 mm thick subsamples were cut from two ends and the middle of each ovary. Subsequently, each sub‐sample was weighed (nearest 0.01 mg), and oocytes were separated with a needle in a petri dish containing tap water. Mature oocytes were counted using a magnifying glass. The relative fecundity was obtained from the formula RF = PF/BW (PF: potential fecundity, BW: fish body weight). The egg diameter was measured from 30 samples at each site using the Motic Images Pro Plus 2.0 software (Dinh et al., 2016).

### 2.3 Data analyses

The values of GSI were not independent as they were collected monthly over time, so circular analyses (Circular package Version 0.5‐0) were performed. The Rayleigh test was used to verify if the GSI values were uniform. The W, TL, GSI, and frequency of gonadal development stages of males and females at four sampling sites were visualized using stacked rose diagrams. All analyses were performed using R version 4.3.2 (R Core Team, 2023).

The GSI variations according to sex and season were quantified using the Mann–Whitney test, and the spatial changes in GSI were confirmed using the Kruskal–Wallis test. If PF, RF, and egg diameter were normally distributed, the Kolmogorov–Smirnov test was applied (Kim, 2015). If their distribution was normal, the Levene test was used to assess for equality of variances, and the *T*‐test was used to determine their differences per site by sex and season. Meanwhile, the Mann–Whitney test was used if they were found to have a non‐normal distribution. The Levene test evaluated the equality of variances between four areas and twelve months. In cases of equal variance, one‐way ANOVA and Tukey's Post Hoc test were used to test the differences of these values at different sampling sites and months; however, one‐way ANOVA and Tamhane's T2 were used for analyses in the case of unequal variance. Similarly, the Kolmogorov–Smirnov test was used to assess whether the PF and RF by sampling sites were normally distributed. If the variation of their values at sampling sites was not normally distributed, the Kruskal–Wallis test was performed; otherwise, one‐way ANOVA was used. Logarithmic regression was used to analyze the correlation between fish body size (TL and *W*) and PF (Metin et al., 2011). SPSS software v.21 was used to analyze all data, and all tests were considered significantly different at the 5% level.

## 3 RESULTS

### 3.1 Sex ratio

**FIGURE 3:**
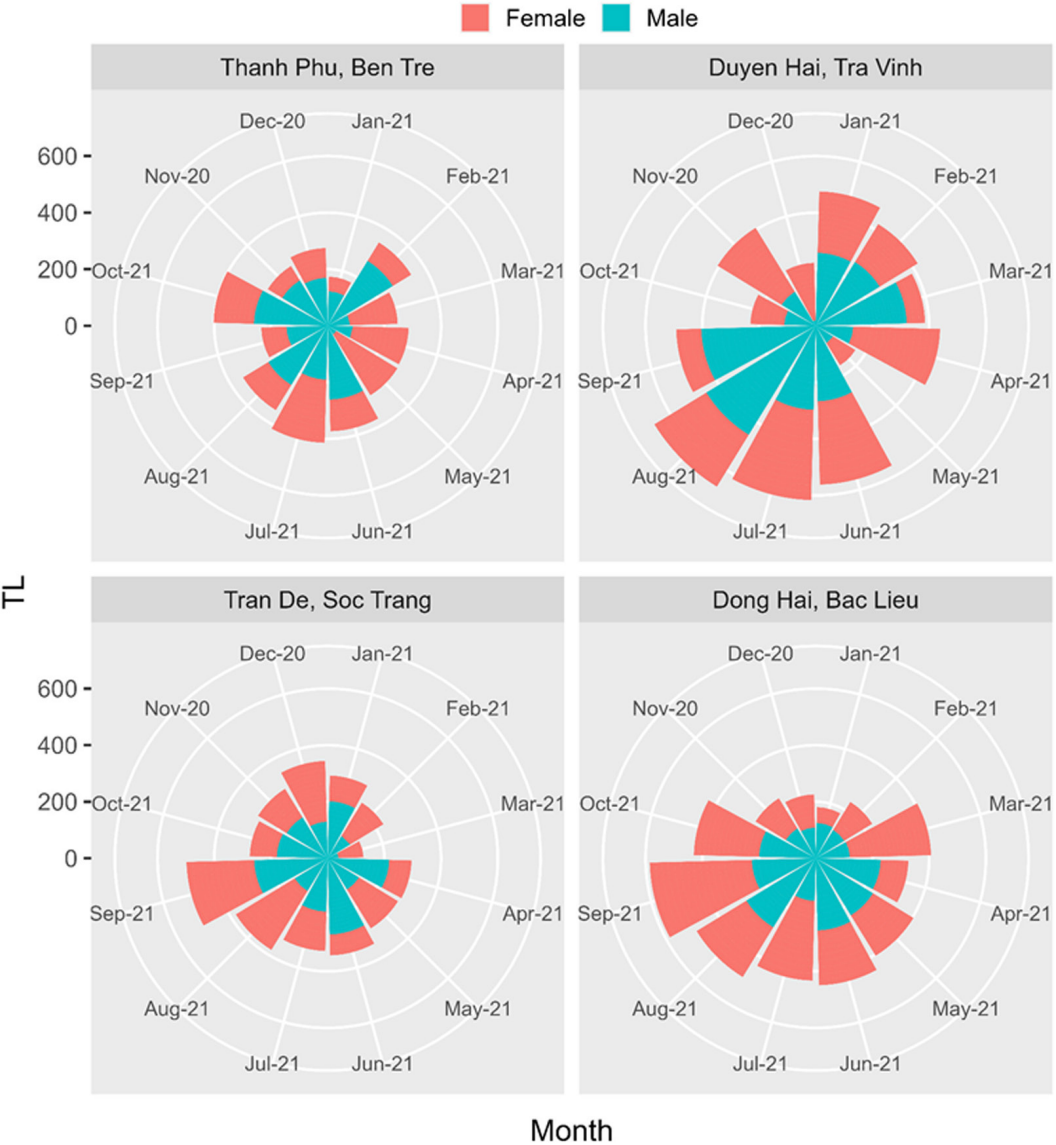
The weight (W) of male and female *Ellochelon vaigiensis* specimens at four sampling sites.

**FIGURE 4:**
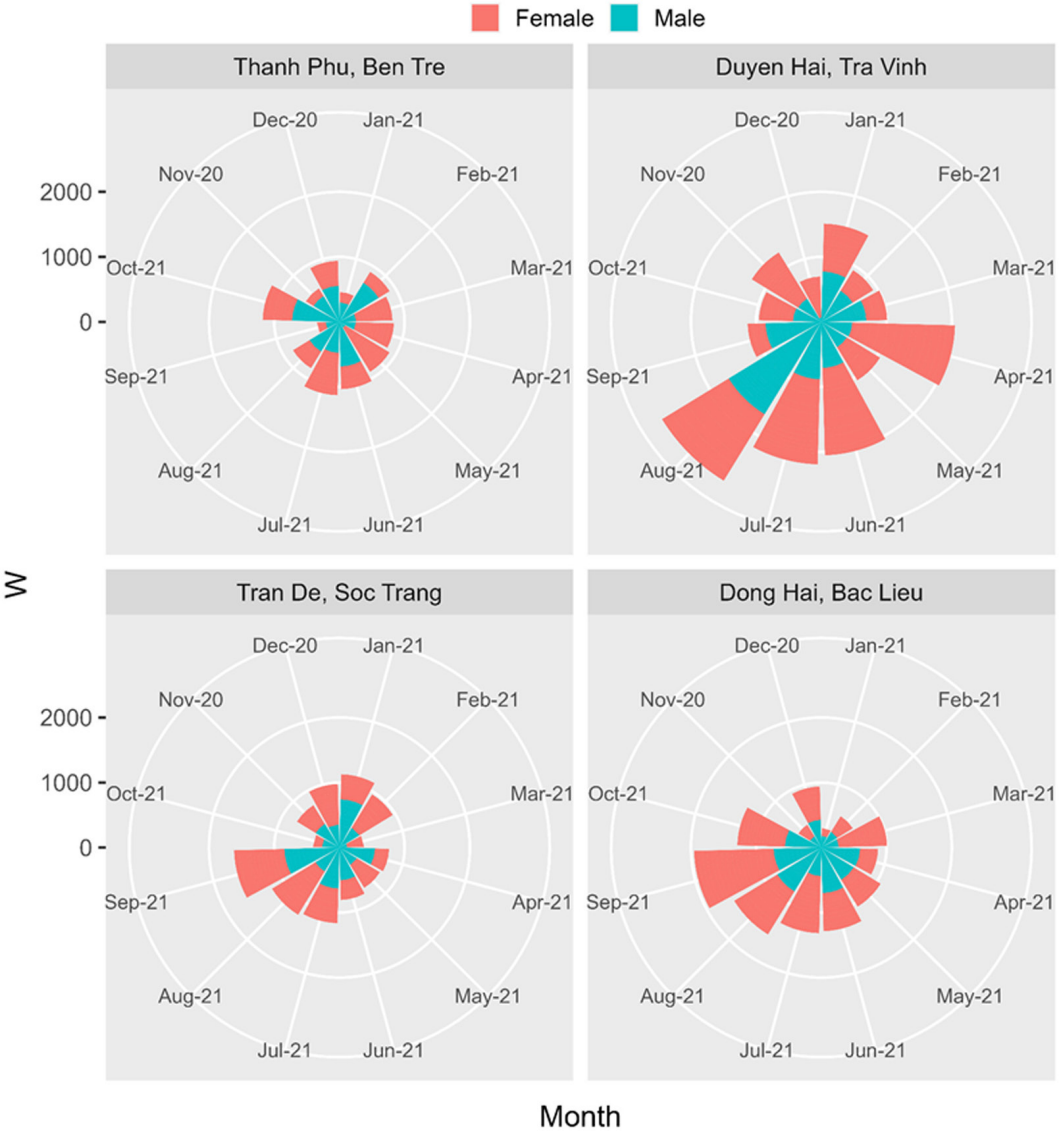
The total length (TL) of male and female *Ellochelon vaigiensis* specimens at four sampling sites.

A total of 942 specimens (526 males and 416 females) of *E. vaigiensis* were obtained from November 2020 to October 2021, with the total length and weight range being 7.6–32.6 cm and 5.5–234.2 g, respectively. The total length and weight of mullets fluctuated monthly at the four sampling sites visualized in Figures 3 and 4, showing the highest values at Duyen Hai, Tra Vinh, corresponding to the longest bars. The overall sex ratio of *E. vaigiensis* from all four sites was 1.26 males: 1.00 females (*χ* ^2^ = 12.85, df = 1, *p* = .00). The females were outnumbered by the males in Thanh Phu, Ben Tre (1.39 males: 1.00 females, *χ* ^2^ = 5.75, df = 1, *p* = .016) and Duyen Hai, Tra Vinh (1.35 males: 1.00 females, *χ* ^2^ = 5.74, df = 1, *p* = .017) but not in Tran De, Soc Trang and Dong Hai, Bac Lieu ($\chi_{\text{TDST}}^{2}$ = 3.49, df = 1, *p* = .062) ($\chi_{\text{DHBL}}^{2}$ = 0.32, df = 1, *p* = .573). In addition, more males were captured in both dry and wet seasons with the sex ratio of 1.23:1.00 ($\chi_{\mathrm{Dry}\;\text{season}}^{2}$ = 3.95, df = 1, *p* = .047) and 1.29:1.00 ($\chi_{\mathrm{Wet}\;\text{season}}^{2}$ = 9.00, df = 1, *p* = .003), respectively.

### 3.2 Spermatogenesis

**FIGURE 5:**
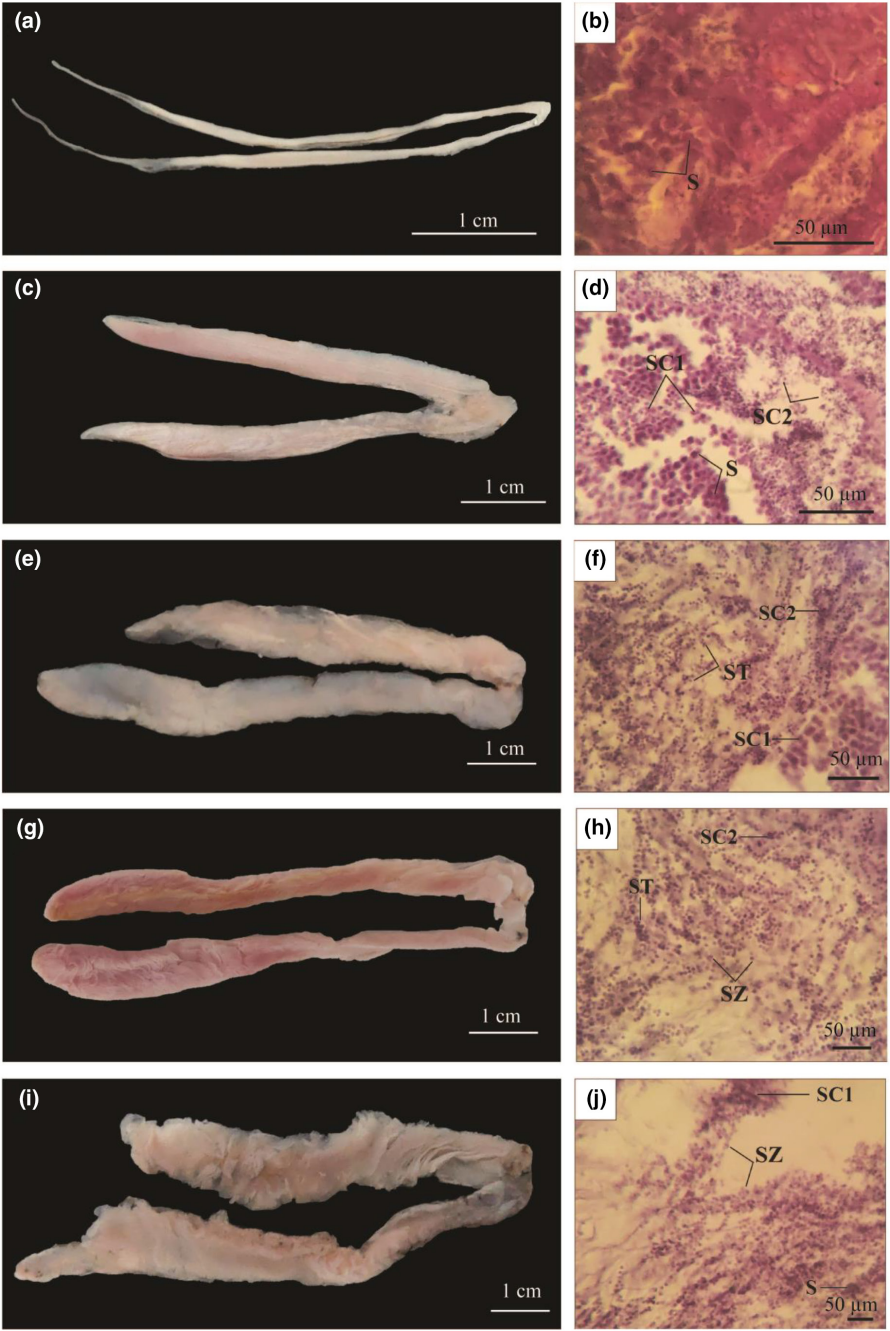
Testicular morphology and histology of *Ellochelon vaigiensis* (a–e: stages I–V; f–j: stages I–V; S: Spermatogonia, SC1: primary spermatocytes, SC2: secondary spermatocytes, ST: spermatid, and SZ: spermatozoa).

In stage I (immature stage), the testes were observed to be paired, elongated, and filiform with a thin peritoneal layer. Testes in stage I were not discernible to the naked eye, so histological slides were stained with hematoxylin and eosin to identify male gonads (Figure 5a). Histological sections showed that testes in stage I contain spermatogonia (S). The S was basophilic and stained dark purple with hematoxylin (Figure 5b). The testes gradually increased and occupied about 1/5 of the abdominal cavity in stage II (developing stage); they are ribbon‐like, pale pink, and sharp with thin edges (Figure 5c). Testicular histology samples at this stage were examined under the microscope. In addition to spermatogonia (S), there was also the presence of primary spermatocytes (SC1) and secondary spermatocytes (SC2). The nuclei of SC1 and SC2 were both strongly stained with hematoxylin and eosin (Figure 5d).

In stage III (maturing stage), testes were wider than in stage II, occupied about 1/3 of the abdominal cavity, were milky white, and ribbon‐like in shape (Figure 5e). The testes were composed entirely of spermatids (ST) at this stage (Figure 5f). At stage IV (mature stage), testes developed to the largest sizes, occupying 2/3 or more of the abdominal cavity, pinkish, wrinkled, and blood irrigation were evident (Figure 5g). Testicular lobules expanded and filled with spermatozoa (SZ). The SZ were very small cells with sphere‐shaped nuclei when stained with hematoxylin (Figure 5h). The testes were creamy and wrinkled in stage V (ripe stage) (Figure 5i). In histological samples, the testes mainly consisted of SZ (Figure 5j). Males with testes in stage VI (recovering stage) were not encountered in this study.

### 3.3 Oogenesis

**FIGURE 6:**
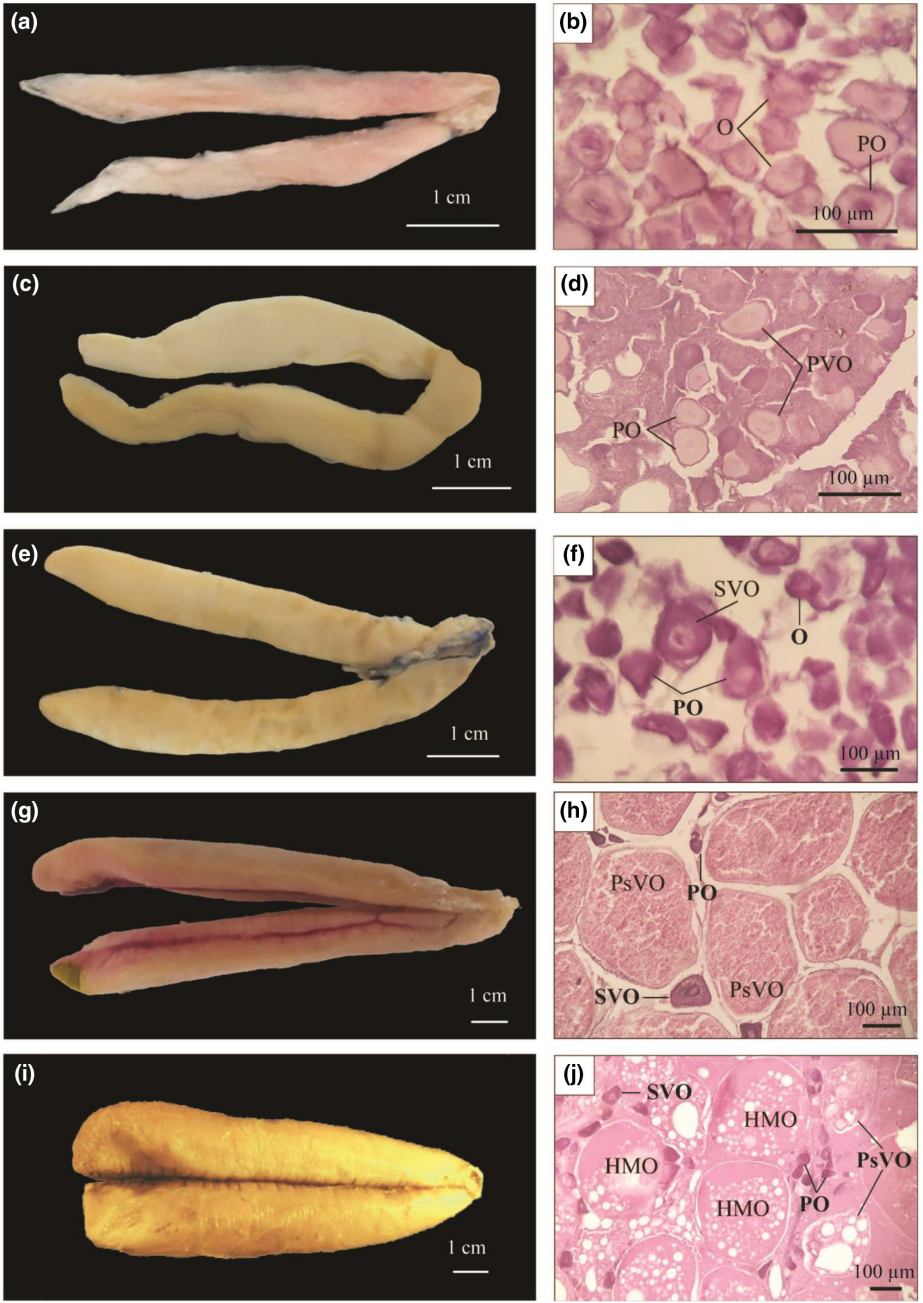
Ovarian morphology and histology of *Ellochelon vaigiensis* (a–e: stages I–V; f–j: stages I–V; GC: germ cells, O: oogonia, PO: primary oocyte, PVO: secondary vitellogenic oocytes, SVO: secondary vitellogenic oocytes, PsVO: post vitellogenic oocytes, HMO: hydrated oocytes).

At stage I (immature stage), ovaries were small, thin, filiform, and transparent (Figure 6a). Oogonia (O) and primary oocytes (PO) were seen primarily in the ovaries. The cytoplasm was strongly basophilic; each nucleus was small, round, and pale purple (Figure 6b). At stage II (growing stage), the ovaries were light yellow, rounder, and larger than in the previous stage, occupying nearly one‐third of the abdominal cavity (Figure 6c). The ovaries comprised primary oocytes (PO) and primary vitellogenic oocytes (PVO) with large nuclei. A few yolk sac granules were found in the cytoplasmic ovary (Figure 6d). At stage III (maturing stage), the ovaries were pale yellow, smooth, and covered with prominent blood vessels (Figure 6e). The ovaries contained only the secondary vitellogenic oocytes (SVO). Nutrients of oocytes were produced in fat droplets and yolk granules, which did not stain with dyes (Figure 6f). Stage III lasted for quite some time; all individuals with stage III ovaries were encountered every month of the year.

At stage IV (mature stage), the ovaries were larger and longer, with protruding and visible blood vessels (Figure 6g). The ovaries occupied ¾ of the abdominal cavity. The egg granules were round, separated from each other easily, pinkish‐red, and could be counted to calculate the batch fecundity. Post vitellogenic oocytes (PsVO) were found in the ovaries in this stage; the nucleus was centrally located, and no nucleolus was observed (Figure 6h).

The ovaries occupied almost the entire abdominal cavity at stage V (ripe stage), reaching the largest size, and appeared yellow, smooth, and turgid (Figure 6i). Eggs were orange‐yellow, round, and easy to separate. The histological sections of the ovaries at this stage contained mainly hydrated oocytes (HMO) and the growing oocytes (Figure 6j). They were ready to be released from the follicles and connective tissue out of the female body. This suggests that *E. vaigiensis* is a synchronic spawner. Females with ovaries in stage VI (recovering stage) were not recorded in this study.

### 3.4 Seasonality and gonadosomatic indices

The monthly GSI values of males and females at four sampling sites were visualized in Figure 6. The Rayleigh test showed that GSI values were not uniform (*p* = .0054). The GSI values of females (2.18 ± 0.16 SE) were always significantly higher than that of males (0.48 ± 0.04 SE) (Mann–Whitney *U*, *Z* = −14.92, df = 1, *p* = .00), and in the wet season, GSI values (1.55 ± 0.12 SE) were also higher than the dry season (0.72 ± 0.09 SE) (*Z* = −8.33, df = 1, *p* = .00).

The GSI values showed differences according to sampling sites (Kruskal–Wallis H, *χ* ^2^ = 16.52, df = 3, *p* = .001) and sampling months (*χ* ^2^ = 121.37, df = 11, *p* = .00). The highest GSI value was recorded in Thanh Phu, Ben Tre (1.44 ± 0.20 SE), and the lowest in Duyen Hai, Tra Vinh (0.97 ± 0.13 SE). The GSI values from January to April (the dry season) were statistically lower than the rest of the months. The GSI value was the highest in July 2021 (2.77 ± 0.44 SE), followed by September and August 2021. The lowest GSI was recorded in April 2021 (0.47 ± 0.14 SE) (Figure 6).

**FIGURE 7:**
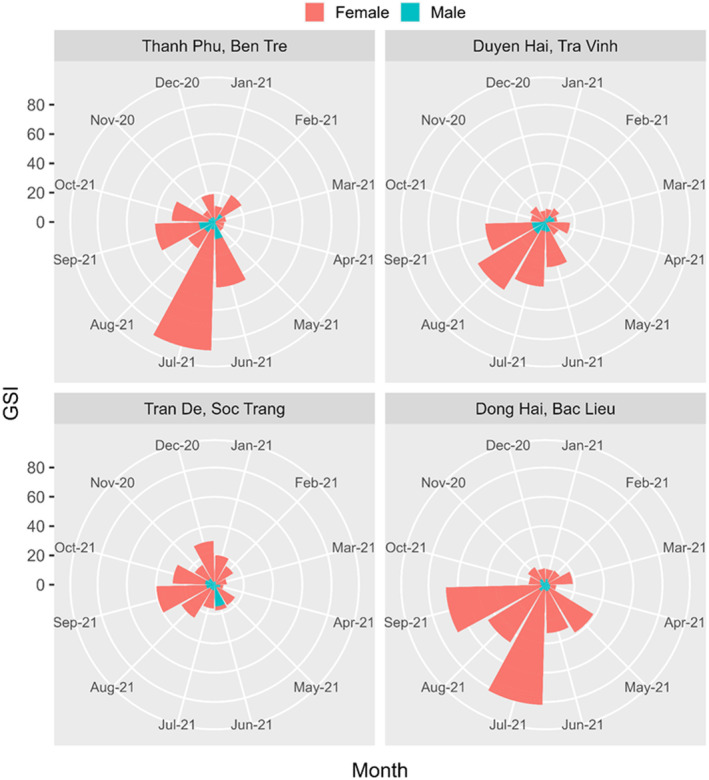
Gonadosomatic indices (GSI) of male and female *Ellochelon vaigiensis* specimens at four sampling sites.

A similar trend was also detected in all GSI values of both sexes at each sampling site. The monthly change trend of GSI of males and females at four sites was given in Figure 7. These GSI values were higher in the wet season than in the dry season for males and females at all four sampling sites. For example, the mean GSI values in males in Duyen Hai, Tra Vinh were high from June to September, decreasing in the remaining months. Another example, GSI values were high for females from May to September in Dong Hai, Bac lieu. Thereafter, there was a decline in the average GSI values from October to April.

**FIGURE 8:**
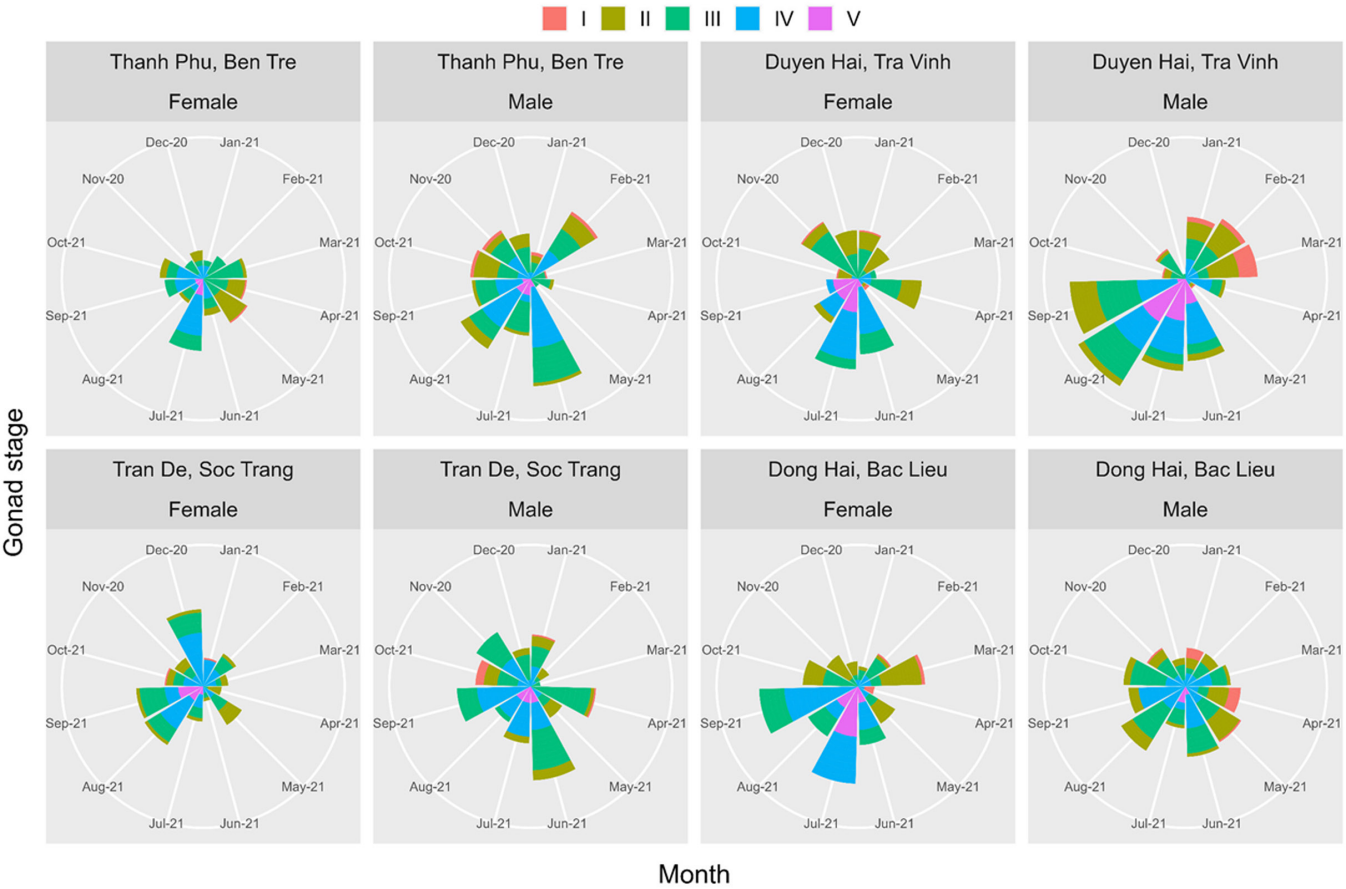
Gonadal stage composition of male and female *Ellochelon vaigiensis* specimens at four sampling sites.

The appearance of mature gonads (stages IV and V) and the fluctuations in GSI values were the basis for predicting the breeding season. Males with mature gonads were found mostly from June to September (Figure 8), coincident with the period when females were most encountered (Figure 8). Moreover, GSI values of both sexes were high in the wet season, particularly from June to September. This indicated that the spawning season of *E. vaigiensis* is in the wet season but peaks from June to September.

### 3.5 Length at maturity, egg diameter, and fecundity

**FIGURE 9:**
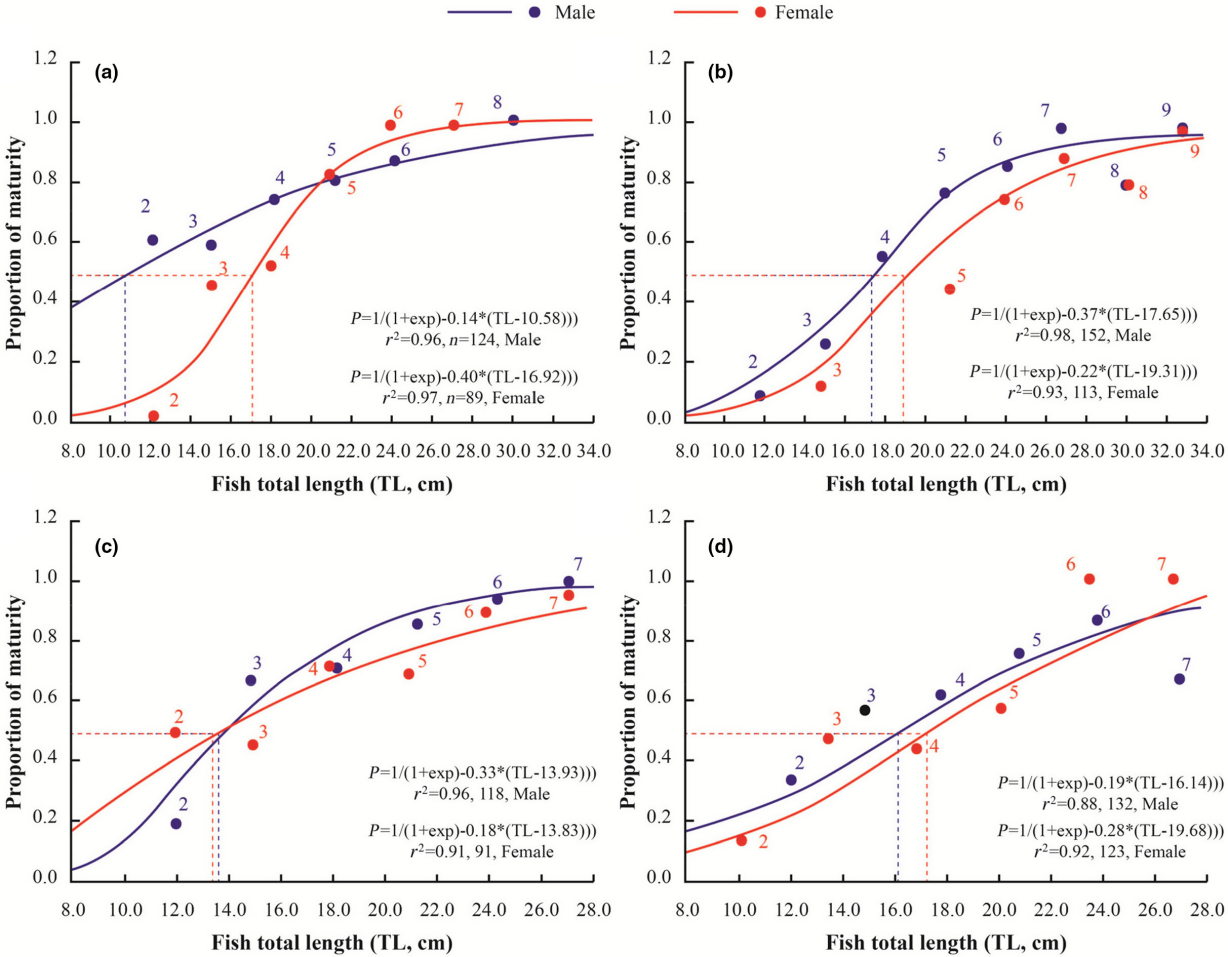
Size at first maturity of male and female *Ellochelon vaigiensis* specimens (a, b, c, and d represent Thanh Phu, Ben Tre; Duyen Hai, Tra Vinh; Tran De, Soc Trang; Dong Hai, Bac Lieu).

The length at maturity (*L*_m_) of *E. vaigiensis* showed the variation between males and females as well as between the four sites (Figure 9). The length at 50% maturity of males and females recorded at Duyen Hai, Tra Vinh (17.7 and 19.3 cm) and Dong Hai, Bac Lieu (16.1 and 19.7 cm) were larger than the other two study sites (Figure 9). Overall, the results suggest that mature males of *E. vaigiensis* are smaller than females.

**FIGURE 10:**
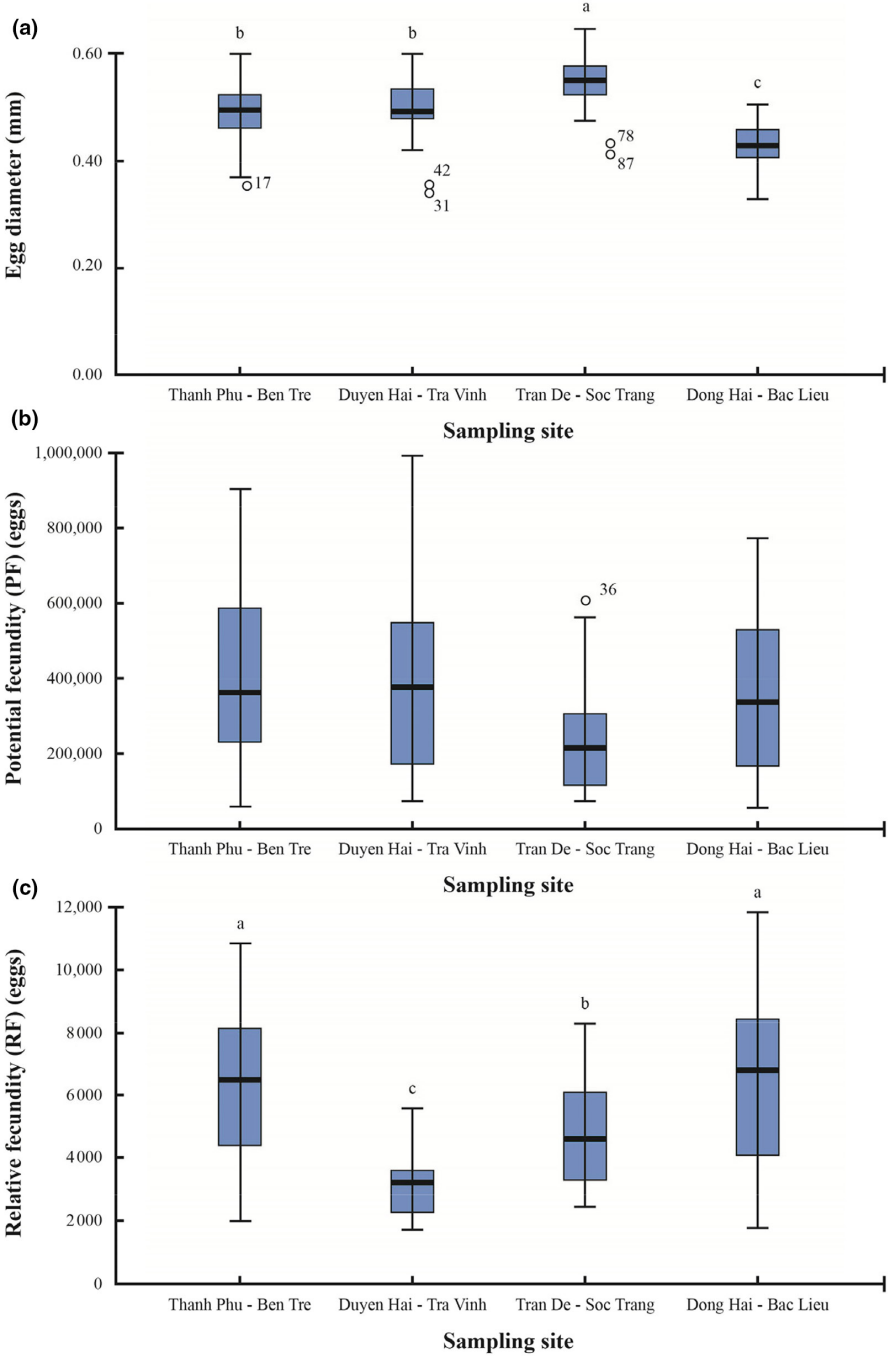
Egg diameter (a), potential fecundity (b), and relative fecundity (c) of *Ellochelon vaigiensis* specimens at four sampling sites.

Figure 10 illustrates the variation of egg diameter, potential, and relative fecundity at four sampling sites. The diameter of the egg showed normal distribution (Kolmogorov–Smirnov test, KS = 0.058, df = 120, *p* = .20) and ranged from 0.43 to 0.54 mm, of which the largest diameter values were found at Duyen Hai, Tra Vinh (One‐way ANOVA, *F* = 25.57, df = 3, *p* = .00, Figure 10a).

Both potential and relative fecundity did not show the normal distribution (Kolmogorov–Smirnov test, KS_F_ = 0.109, KS_RF_ = 0.124, df = 80, *p* = .001 in two cases). The potential fecundity was not different between sampling sites (Kruskal–Wallis H Test, *χ* ^2^ = 6.65, df = 3, *p =* .08, Figure 10b) and fluctuated from 246,254 ± 35,878 to 411,970 ± 54,370 eggs. On the contrary, the relative fecundity showed the variation at each sampling site (*χ* ^2^ = 22.29, df = 3, *p* = .00, Figure 10c), namely, the highest value was found in Thanh Phu, Ben Tre (6388 ± 605 eggs) and Dong Hai, Bac Lieu (6461 ± 637 eggs), and 3117 ± 223 eggs were the lowest value in Duyen Hai, Tra Vinh.

**FIGURE 11:**
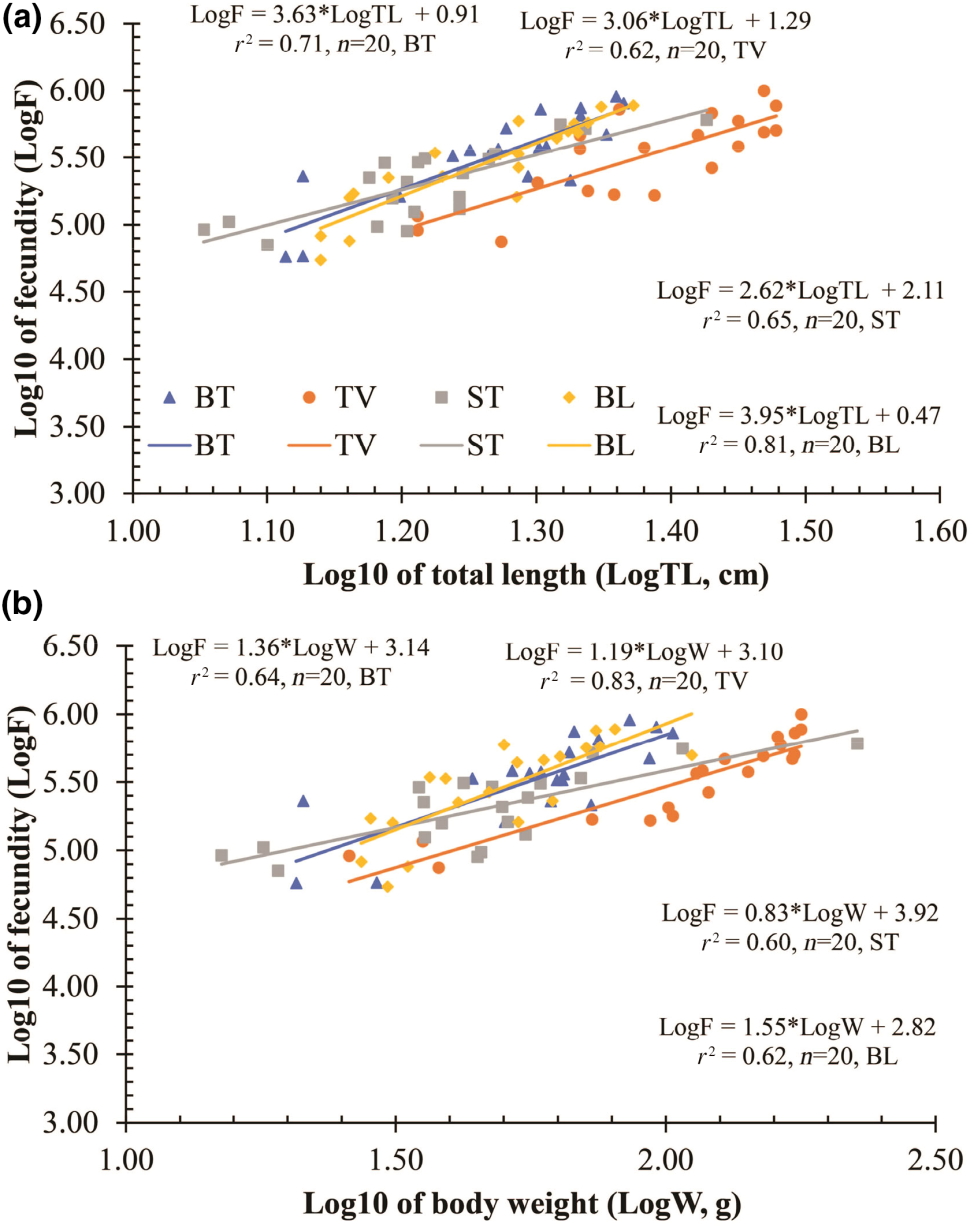
Relationships between fecundity and total length (a) and body weight (b) of *Ellochelon vaigiensis* specimens (BT: Thanh Phu, Ben Tre; TV: Duyen Hai, Tra Vinh; ST: Tran De, Soc Trang; BL: Dong Hai, Bac Lieu).

The potential fecundity exhibited a proportional relationship with the total length (TL) and body weight (*W*) of males and females since the *r* ^2^ values of the relationships between PF and fish size were higher than 0.60 (Figure 11).

## 4 DISCUSSION

Our study shows a significant dominance of males at the study sites. Knowledge of the sex ratio of fish species is important in assessing the reproductive potential of populations (Vazzoler, 1996a). Wootton (1990a) emphasized that differences in mortality rate, development, life span, the activities of sex, and migration across reproductive regions can result in the dominance of one of the sexes. The overall sex ratio in this study was 1.26 males:1.00 females. More males may be indicative of fishing activities targeting larger individuals (such as females) (Dinh, Truong, Nguyen, Tran, et al., 2022), and females tend to accumulate more fat than males. At Tran De, Soc Trang and Dong Hai, Bac Lieu, the sex ratios were approximately 1:1, different from the other two study sites. The difference in sex ratio between sites can be due to different environmental conditions, especially substrate, available organic matter, and sampling method, which ultimately affects the sex ratio (Trisyani, 2018). According to Maskill et al. (2017), the sex ratio did not significantly affect the success of the fertilization process. The reproductive success of fish species depended on the number of gametes. The more males a population has, the more gametes are produced, or vice versa, depending on the sex ratio (Coscia et al., 2016). In studied populations of *Mugil*, the number of females were more than that of males. For example, the sex proportion in *Mugil cephalus* was 0.88 males: 1 females in the Central Mexican Pacific Coast (Elaine et al., 2016) and 1.00 males:1.10 females in Tamiahua Lagoon, Mexico (Aguirre & Gallardo‐Cabello, 2004). The sex proportion of *M. curema* was 1.00:1.40 in Mexico (Aguirre & Gallardo‐Cabello, 2004), 1.00:2.40 in southeastern Brazil (Fernandez & Dias, 2013), and 0.74:1.00 in Pernambuco (Santana, 2007). Similarly, for *Planiliza abu*, the overall sex ratio was 1.00:1.04 male: female of samples from Atatürk Dam Lake, southeastern Turkey (Şahinöz et al., 2011); and 1.00:1.21 in the Tigris River (Unlü et al., 2000).

Histological analyses of ovaries in squaretail mullets from the VMD revealed two types of oocytes found simultaneously in individual females—the growth and the mature stages—suggesting that *E. vaigiensis* is a synchronic spawner (see Wallace & Selman, 1981). Similar reproduction patterns were found in *M. curema* (Fernandez & Dias, 2013; Santana, 2007) and *Mugil platanus* (González‐Castro et al., 2011). This spawning strategy is hypothesized to maximize offspring fertility concerning the energy availability and longevity of the broodfish (Roff, 1992).

The gonadosomatic index values of both sexes in *E. vaigiensis* were higher in the wet season than in the dry season at all four sampling sites. Variations over time in GSI values may indicate periods of peak reproductive activity for the species (Wootton, 1990b). In the present study, the high GSI values concentrated in the wet season at all four sites suggested that squaretail mullet spawned in the wet season. This conclusion was again confirmed by the frequency of occurrence of mature gonads. Specifically, the mature testes and ovaries were more frequently encountered in the wet season and peaked from June to September 2021. In short, the spawning season of *E. vaigiensis* in the estuaries of VMD was in the wet season, with an intensive period from June to September. The onset of the spawning season for different fish species is influenced primarily by environmental factors (Vazzoler, 1996b). The spawning season of varying fish species typically coincides with the wet season likely because of the abundance and availability of nutrient resources due to the high rainfall (Blaber, 2000; Elliott et al., 2007; Whitfield, 1990). However, in the research of Grant and Spain (1975), the breeding season of *E. vaigiensis* from tropical Australia fell during the summer monsoon period; females reached sexual maturity in September and began spawning in February and March. According to Chen et al. (1999), the variation in the spawning season of mullet species depended on geographical conditions and environmental factors. For example, the spawning season of *L. macrolepis* in Southern Taiwan was from December to May, coinciding with periods of decreased daylight hours (Chen et al., 1999); in Sri Lanka, in January–February and August–September (Wijeyaratne & Costa, 1987); and in India in July–August (Luther, 1963). Another example in the Canane'ia‐Iguape and Santos estuaries, the coast of São Paulo, Brazil, the spawning of *M. curema* occurred at two time periods: April and November with higher intensity, at the beginning and end of the wet season, respectively (Fernandez & Dias, 2013). However, in the northeastern region of Brazil, the breeding season of the same species occurs from November to March (Luchiari, 2011). In the Mar Chiquita, Argentina, *Mugil platanus* also spawned in two time periods: April–May and November–December (González‐Castro et al., 2011).

The average length at 50% maturity (*L*_m_) in four sample collection sites demonstrate that mature females of *E. vaigiensis* are longer than males. According to Pauly (2021), females grow faster than males because they are less active, so they use less oxygen than males (about 90% of the oxygen the fish receives from the gills is used for various activities, and the rest is for growth). Growth in length of square tail mullet was superior in Duyen Hai, Tra Vinh, and Dong Hai, Bac Lieu, where there was a rich mangrove ecosystem with a salinity of about 10.4‰ and 16.7‰. In teleost fishes, the larger and older females tend to lay more and larger eggs (Chambers & Leggett, 1996; Palumbi, 2004). Larger males are more successful at mating as they tend to succeed in territorial or dominance displays (Warner, 1988). Thus, the growth rate can also be an important determinant of reproductive success (Coscia et al., 2016). This outcome was consistent with previous reports of González‐Castro et al. (2011) on *M. platanus* (45.0 cm in females and 43.6 cm in males); McDonough et al. (2005) on *M. cephalus* in South Carolina (32.5 cm in females and 27.5 cm in males); and Kendall and Gray (2008) on *Liza argentea* in southeastern Australia (20.7 cm in females and 18.0 cm in males). Nevertheless, in other studies, female and male mullets had approximately equal *L*_m_ values, similar to another mullet species, *M. cephalus*, from a coastal lagoon in Mexico (Aguirre & Gallardo‐Cabello, 2004); and *Liza abu* from the Tigris River (Turkey) (Unlü et al., 2000). Fernandez and Dias (2013) noted that differences in the length of maturity for one species might be commonly related to the geographic position of the surveyed populations.

The potential fecundity of *E. vaigiensis* was 246,254–411,970 eggs for female individuals with a mean TL of 26.7–32.6 cm and *W* of 102.97–234.19 g. The relative fecundity of mullet varied according to sampling sites, possibly due to their interaction and adaptation to natural conditions in their habitat. The higher fertility was recorded in Dong Hai‐Bac Lieu and Thanh Phu‐Ben Tre, corresponding to the highest and lowest salinity values (16.7‰ and 8.2‰). Mullets are widely distributed fishes with large numbers and high biomass in coastal ecosystems where they occur. They exhibit flexible physiological characteristics but are typically near the base of aquatic food webs. Salinity is an important factor influencing the abundance of different mullet species in estuaries. Each species has optimal salinity conditions for food digestion and assimilation (Whitfield, 2015). This study shows that *E. vaigiensis* can adapt to a wide water salinity spectrum. These values were relatively close to those found in other mullet species, such as *Liza aurata* from Iranian waters (452,000 eggs) (Fazli et al., 2008) and *L. argentea* from southeastern Australia (321,260 eggs) (Kendall & Gray, 2008). Meanwhile, some species of mullet exhibited higher potential fecundity, for example, *E. vaigiensis* (805,000 and 1,204,000 eggs) (Grant & Spain, 1975), *M. platanus* (1,800,000 eggs of average) González‐Castro et al. (2011), *M. cephalus* (540,706–1,483,056) (Aguirre & Gallardo‐Cabello, 2004), and *Mugil liza* (2,040,000–3,650,000) (Albieri et al., 2010). The PF was species‐specific and changed depending on habitat conditions, indicating that fishes can adapt to different habitat conditions to optimize fertility. For example, the potential and relative fecundities of *M. curema* in Veracruz, Mexico were 51,901–346,701 and 1064 (Aguirre & Gallardo‐Cabello, 2004), while in Rio de Janeiro, Brazil, they were 123,000–711,000 and 750.54 (Albieri et al., 2010), and in Margarita Island, Venezuela PF was 190,000–1,040,000 (Marin et al., 2003). In contrast, the relative fecundity (*RF*) value of *E. vaigiensis* represented a statistically significant difference at each of the four sampling sites. Specifically, the highest *RF* values were recorded at Thanh Phu – Ben Tre and Dong Hai – Bac Lieu, where salinity values were lowest and highest, respectively 8.2‰ and 16.7‰.

There was an increase in the number of eggs laid by larger fishes in Ben Tre, Tra Vinh, Soc Trang, and Bac Lieu, based on total length (TL) and body weight (*W*). Strong correlations were discovered between egg numbers with TL (*r* ^2^ = 0.65) and *W* (*r* ^2^ = 0.68) in *L. abu* from the Tigris River, Turkey (Unlü et al., 2000). The potential fecundity of *M. curema* from coastal systems in southeastern Brazil strongly correlated with TL (*r* ^2^ = 0.812) and *W* (*r* ^2^ = 0.617). In the study, high values of *r* ^2^ in *E. vaigiensis* between PF and fish size (*r* ^2^ > 0.6) were compatible with the above results. This indicated that the larger the female, the more eggs she produces (Chambers & Leggett, 1996; Palumbi, 2004). Jonsson and Jonsson (1997) suggested that larger female fishes have larger body cavities to accommodate more eggs. This proportional relationship may be due to the enlargement of the abdominal cavity in females during the growth period, along with the possible increase in gonadal size, and this increase in fertility peaked with gonadal maturation and reduced in older individuals (Fernandez & Dias, 2013).

## 5 CONCLUSION

*Ellochelon vaigiensis* is found to be a synchronic spawner within the VMD, with the breeding season occurring throughout the wet season but intensifying from June to September. Therefore, fishing should be limited during the spawning period to manage future fisheries and ensure the sustainability of this species. In the population, the females were less than males, with a ratio of 1.26 males: 1.00 females. However, females were considerably longer at maturity than males. We recommend to only harvest fishes that are 10 cm (the length at 50% maturity ranged from 10.6 to 19.3 cm). The *RF* differed by sites, indicating that *E. vaigiensis* is adapted to different salinity regimes.

## AUTHOR CONTRIBUTIONS

**Quang Minh Dinh:** Conceptualization (equal); funding acquisition (equal); investigation (equal); methodology (equal); project administration (lead); writing – original draft (equal); writing – review and editing (equal). **Ngon Trong Truong:** Conceptualization (equal); investigation (equal); writing – original draft (equal); writing – review and editing (equal). **Ton Huu Duc Nguyen:** Investigation (equal); methodology (equal); writing – original draft (equal); writing – review and editing (equal). **Tran Thi Huyen Lam:** Conceptualization (equal); funding acquisition (equal); investigation (equal); writing – original draft (equal); writing – review and editing (equal). **Tien Thi Kieu Nguyen:** Funding acquisition (equal); investigation (equal); writing – original draft (equal); writing – review and editing (equal). **Zeehan Jaafar:** Conceptualization (equal); funding acquisition (equal); investigation (equal); writing – original draft (equal); writing – review and editing (equal).

## FUNDING INFORMATION

This work was funded by VINGROUP and supported by Vingroup Innovation Foundation (VINIF) under project code VINIF.2020.DA01.

## CONFLICT OF INTEREST STATEMENT

The authors declare that they have no competing interests.

## Supporting information


Data S1.

## Data Availability

Data were uploaded to the journal system as Supporting Information for review and publication.

## References

[ece311033-bib-0001] Aguirre, A. L. I. , & Gallardo‐Cabello, M. (2004). Reproduction of *Mugil cephalus* and *M. curema* (Pisces: Mugilidae) from a coastal lagoon in the Gulf of Mexico. Bulletin of Marine Science, 75(1), 37–49.

[ece311033-bib-0002] Albieri, R. J. , Araújo, F. G. , & Uehara, W. (2010). Differences in reproductive strategies between two co‐occurring mullets *Mugil curema* Valenciennes 1836 and *Mugil liza* Valenciennes 1836 (Mugilidae) in a tropical bay. Tropical Zoology, 23(1), 51–62.

[ece311033-bib-0003] Alonso‐Fernández, A. , Alós, J. , Grau, A. , Domínguez‐Petit, R. , & Saborido‐Rey, F. (2011). The use of histological techniques to study the reproductive biology of the hermaphroditic Mediterranean fishes *Coris julis*, *Serranus scriba*, and *Diplodus annularis* . Marine and Coastal Fisheries, 3(1), 145–159. 10.1080/19425120.2011.556927

[ece311033-bib-0004] Bagenal, T. B. (1967). A short review of fish fecundity. In S. D. Gerking (Ed.), The biological basis of freshwater fish production (pp. 89–111). John Wiley.

[ece311033-bib-0005] Ben Khemis, I. , Hamza, N. , & Sadok, S. (2019). Nutritional quality of the fresh and processed grey mullet (Mugilidae) products: A short review including data concerning fish from freshwater. Aquatic Living Resources, 32, 2.

[ece311033-bib-0006] Bennett, W. A. , & Beitinger, T. L. (1997). Temperature tolerance of the sheepshead minnow, *Cyprinodon variegatus* . Copeia, 1997, 77–87.

[ece311033-bib-0007] Blaber, S. J. M. (2000). Tropical estuarine fishes: Ecology, exploitation and conservation. Blackwell Science.

[ece311033-bib-0008] Bledsoe, G. , Bledsoe, C. , & Rasco, B. (2003). Caviars and fish roe products. Critical Reviews in Food Science and Nutrition, 43, 317–356. 12822675 10.1080/10408690390826545

[ece311033-bib-0009] Brian, W. C. (2017). Review of the freshwater mullets of Iran (family Mugilidae). Iranian Journal of Ichthyology, 4(2), 75–130. 10.22034/iji.v4i2.218

[ece311033-bib-0010] Cardona, L. (2016). Food and feeding of Mugilidae. In D. Crosetti & S. J. M. Blaber (Eds.), Biology, ecology and culture of mullets (Mugilidae) (pp. 165–195). CRC Press.

[ece311033-bib-0011] Carleton, H. M. , Drury, R. A. B. , & Wallington, E. (1980). Carleton's histological technique. Oxford University Press.

[ece311033-bib-0012] Chambers, R. C. , & Leggett, W. C. (1996). Maternal influences on variation in egg sizes in temperate marine fishes. American Zoologist, 36(2), 180–196.

[ece311033-bib-0013] Chen, M.‐H. , Wen, D.‐J. , & Chen, C.‐Y. (1999). Reproduction and estuarine utilization of the grey mullet, *Liza macrolepis* (Smith, 1846), in the area of Kaohsiung harbor, southern Taiwan. Fisheries Science, 65(1), 1–10.

[ece311033-bib-0014] Chidambaram, K. , & Kuriyan, G. (1952). Notes on the grey mullets (*Mugil* spp.) of Krusadai Island, gulf of Mannar. Journal of the Bombay Natural History Society, 50(3), 515–519.

[ece311033-bib-0015] Coscia, I. , Chopelet, J. , Waples, R. S. , Mann, B. , & Mariani, S. (2016). Sex change and effective population size: Implications for population genetic studies in marine fish. Heredity, 117(4), 251–258. 27507184 10.1038/hdy.2016.50 PMC5026757

[ece311033-bib-0016] Dinh, Q. M. (2018). Aspects of reproductive biology of the red goby *Trypauchen vagina* (Gobiidae) from the Mekong Delta. Journal of Applied Ichthyology, 34(1), 103–110. 10.1111/jai.13521

[ece311033-bib-0017] Dinh, Q. M. , & Le, T. T. M. (2017). Reproductive traits of the duckbill sleeper *Butis butis* (Hamilton, 1822). Zoological Science, 24(5), 452–458. 10.2108/zs170013 28990470

[ece311033-bib-1020] Dinh, Q. M. , & Nguyen, T. T. D. (2022). Procedure for performing a fixed microscopic specimen of the gonads of fish. Veterinary Integrative Sciences, 20(3), 645–657. 10.12982/vis.2022.049

[ece311033-bib-0018] Dinh, Q. M. , Nguyen, T. H. D. , Truong, N. T. , & Nguyen, T. T. K. (2023). Population biology of *Ellochelon vaigiensis* (Quoy & Gaimard, 1825) in the Mekong Delta, Vietnam. PeerJ, 11, e14901. 10.7717/peerj.14901 36846463 PMC9951797

[ece311033-bib-0019] Dinh, Q. M. , Qin, J. G. , Dittmann, S. , & Tran, D. D. (2016). Reproductive biology of the burrow dwelling goby *Parapocryptes serperaster* . Ichthyological Research, 63(3), 324–332. 10.1007/s10228-015-0502-7

[ece311033-bib-0020] Dinh, Q. M. , Tran, L. T. , Ngo, N. C. , Pham, T. B. , & Nguyen, T. T. K. (2020). Reproductive biology of the unique mudskipper *Periophthalmodon septemradiatus* living from estuary to upstream of the Hau River. Acta Zoologica, 101(2), 206–217. 10.1111/azo.12286

[ece311033-bib-0021] Dinh, Q. M. , Truong, N. T. , Nguyen, T. H. D. , Lam, T. T. H. , Nguyen, T. T. K. , Le, D. Q. , & Das, S. K. (2022). Feeding ecology of *Ellochelon vaigiensis* (Quoy & Gaimard, 1825) living in the Mekong Delta, Vietnam. Ecology and Evolution, 12(9), e9352. 10.1002/ece3.9352 36188496 PMC9490149

[ece311033-bib-0022] Dinh, Q. M. , Truong, N. T. , Nguyen, T. H. D. , Tran, L. T. H. , Nguyen, T. T. K. , & Phan, L. H. (2022). Variations in length‐weight relationship, growth and body condition of the commercial mullet *Ellochelon vaigiensis* in the Vietnamese Mekong Delta. Heliyon, 8(11), e11789. 10.1016/j.heliyon.2022.e11789 36468124 PMC9713347

[ece311033-bib-0023] Elaine, E.‐B. , Gallardo‐Cabello, M. , Puente‐Gomez, M. , & Garcia‐Boa, A. (2016). Reproduction of *Mugil cephalus* (Percoidei: Mugilidae) off the Central Mexican Pacific Coast. Fisheries and Aquaculture Journal, 7(4), 1–9.

[ece311033-bib-0024] Elliott, M. , Whitfield, A. K. , Potter, I. C. , Blaber, S. J. M. , Cyrus, D. P. , Nordlie, F. G. , & Harrison, T. D. (2007). The guild approach to categorizing estuarine fish assemblages: A global review. Fish and Fisheries, 8(3), 241–268. 10.1111/j.1467-2679.2007.00253.x

[ece311033-bib-0025] El‐Marakby, H. , Eid, A. , Abdelghany, A. , & Abdel‐Tawwab, M. (2006). The impact of striped mullet, *Mugil cephalus* on natural food and phytoplankton selectivity at different feeding regimes in earthen fishponds. Journal of Fishery Aquatic Science, 1(1), 87–96.

[ece311033-bib-0026] Eme, J. , Dabruzzi, T. F. , & Bennett, W. A. (2011). Thermal responses of juvenile squaretail mullet (*Liza vaigiensis*) and juvenile crescent terapon (*Terapon jarbua*) acclimated at near‐lethal temperatures, and the implications for climate change. Journal of Experimental Marine Biology and Ecology, 399(1), 35–38.

[ece311033-bib-0027] FAO . (2018). FAO‐fisheries and aquaculture information and statistics branch. Food and Agriculture Organization of the United Nations.

[ece311033-bib-0028] Fazli, H. , Janbaz, A. A. , Taleshian, H. , & Bagherzadeh, F. (2008). Maturity and fecundity of golden grey mullet (*Liza aurata* Risso, 1810) in Iranian waters of the Caspian Sea. Journal of Applied Ichthyology, 24(5), 610–613.

[ece311033-bib-0029] Fernandez, W. S. , & Dias, J. F. (2013). Aspects of the reproduction of *Mugil curema* Valenciennes, 1836 in two coastal systems in southeastern Brazil. Tropical Zoology, 26(1), 15–32.

[ece311033-bib-0030] Frank, K. T. , & Leggett, W. C. (1994). Fisheries ecology in the context of ecological and evolutionary theory. Annual Review of Ecology and Systematics, 25(1), 401–422.

[ece311033-bib-0031] González‐Castro, M. , Macchi, G. J. , & Cousseau, M. B. (2011). Studies on reproduction of the mullet *Mugil platanus* Günther, 1880 (Actinopterygii, Mugilidae) from the mar Chiquita coastal lagoon, Argentina: Similarities and differences with related species. The Italian Journal of Zoology, 78(3), 343–353.

[ece311033-bib-0032] Grant, C. , & Spain, A. (1975). Reproduction, growth and size allometry of *Liza vaigiensis* (Quoy & Gaimard) (Pisces: Mugilidae) from North Queensland inshore waters. Australian Journal of Zoology, 23(4), 475–485.

[ece311033-bib-0033] Hong, P. N. , & San, H. T. (1993). Mangroves of Vietnam (Vol. 7). The IUCN Wetlands Programme.

[ece311033-bib-0034] Hunter, J. R. , Lo, N. C. H. , & Leong, R. J. H. (1985). Batch fecundity in multiple spawning fishes. NOAA Technical Report NMFS, 36, 67–77.

[ece311033-bib-0035] Jonsson, N. , & Jonsson, B. (1997). Energy allocation in polymorphic brown trout. Functional Ecology, 11(3), 310–317.

[ece311033-bib-0036] Kendall, B. W. , & Gray, C. A. (2008). Reproductive biology of two co‐occurring mugilids, *Liza argentea* and *Myxus elongatus*, in south‐eastern Australia. Journal of Fish Biology, 73(4), 963–979.

[ece311033-bib-0037] Kim, T. K. (2015). T test as a parametric statistic. Korean Journal of Anesthesiology, 68(6), 540–546. 10.4097/kjae.2015.68.6.540 26634076 PMC4667138

[ece311033-bib-0038] Le, T. , Nguyen, M. T. , Nguyen, V. P. , Nguyen, D. C. , Pham, X. H. , Nguyen, T. S. , Hoang, V. C. , Hoang, P. L. , Le, H. , & Dao, N. C. (2006). Provinces and City in the Mekong Delta. In T. Le (Ed.), Geography of provinces and cities in Vietnam (Vol. VI, pp. 49–94). Vietnam Education Publishing House.

[ece311033-bib-0039] Luchiari, A. C. (2011). Some aspects of the biology of white mullet, *Mugil curema* (Osteichthyes, Mugilidae), in the northeastern region, Brazil. Pan‐American Journal of Aquatic Sciences, 6(2), 138–147.

[ece311033-bib-0040] Luther, G. (1963). Some observations on the biology of *Liza macrolepis* (Smith) and *Mugil cephalus* Linnaeus (Mugilidae) with notes on the fishery of grey mullets near Mandapam. Indian Journal Of Fisheries, 10(2), 642–666.

[ece311033-bib-0041] Marin, E. B. J. , Quintero, A. , Bussiere, D. , & Dodson, J. J. (2003). Reproduction and recruitment of white mullet (Mugil curema) to a tropical lagoon (Margarita Island, Venezuela) as revealed by otolith microstructure .

[ece311033-bib-0042] Maskill, P. A. , Miller, I. R. , Halvorson, L. J. , Treanor, H. B. , Fraser, C. W. , & Webb, M. A. (2017). Role of sex ratio and density on fertilization success of intensively cultured endangered woundfin. Journal of Fish and Wildlife Management, 8(1), 249–254.

[ece311033-bib-0043] McDonough, C. J. , Roumillat, W. A. , & Wenner, C. A. (2005). Sexual differentiation and gonad development in striped mullet (*Mugil cephalus* L.) from South Carolina estuaries. Fishery Bulletin, 103(4), 601–619.

[ece311033-bib-0044] Metin, G. , Ilkyaz, A. T. , Soykan, O. , & Kinacigil, H. T. (2011). Age, growth and reproduction of four‐spotted goby, *Deltentosteus quadrimaculatus* (Valenciennes, 1837), in İzmir Bay (central Aegean Sea). Turkish Journal of Zoology, 35(5), 711–716.

[ece311033-bib-0045] Mondal, A. , Chakravortty, D. , Mandal, S. , Bhattacharyya, S. , & Mitra, A. (2015). Feeding ecology and prey preference of grey mullet, *Mugil cephalus* (Linnaeus, 1758) in extensive brackish water farming system. Journal of Marine Science Research & Development, 6(1), 1–5. 10.4172/2155-9910.1000178

[ece311033-bib-0046] Morita, K. , & Morita, S. H. (2002). Rule of age and size at maturity: Individual variation in the maturation history of resident white‐spotted charr. Journal of Fish Biology, 61(5), 1230–1238.

[ece311033-bib-0047] Nelson, J. , Grande, T. , & Wilson, M. (2016). Fishes of the world. John Wiley & Sons.

[ece311033-bib-0048] Nguyen, T. H. D. , Nguyen, H. T. T. , Tran, T. C. , Nguyen, Y. T. N. , & Dinh, Q. M. (2020). Morphometric and meristic variations of *Glossogobius sparsipapillus* along the coastline in the Mekong Delta, Vietnam. International Journal of Zoology and Animal Biology, 3(1), 1–9. 10.23880/izab-16000211

[ece311033-bib-0049] Nguyen, T. M. A. , Nguyen, H. D. T. , & Dinh, M. Q. (2022). Morphological characteristics of digestive tract and clark index of *Ellochelon vaigiensis* (Quoy & Gaimard, 1825) in some coastal estuarine areas in the Mekong Delta. VNU Journal of Science: Natural Sciences and Technology, 38(3), 97–103. 10.25073/2588-1140/vnunst.5456.

[ece311033-bib-0050] Palumbi, S. R. (2004). Why mothers matter. Nature, 430(7000), 621–622. 15295581 10.1038/430621a

[ece311033-bib-0051] Pauly, D. (2021). The gill‐oxygen limitation theory (GOLT) and its critics. Science Advances, 7(2), eabc6050. 33523964 10.1126/sciadv.abc6050 PMC7787657

[ece311033-bib-0052] R Core Team . (2023). R: A language and environment for statistical computing. R Foundation for Statistical Computing.

[ece311033-bib-0053] Rahman, M. A. U. , Lyla, P. , & Khan, S. A. (2016). Food and feeding habits of the greenback grey mullet *Liza subviridis* (Valenciennes, 1836) from Parangipettai waters, south‐east coast of India. Indian Journal Of Fisheries, 63(4), 126–131. 10.21077/ijf.2016.63.4.60271-20

[ece311033-bib-0054] Roff, D. (1992). Evolution of life histories: Theory and analysis. Springer Science & Business Media.

[ece311033-bib-0055] Roff, D. A. (1981). Reproductive uncertainty and the evolution of iteroparity: Why don't flatfish put all their eggs in one basket? Canadian Journal of Fisheries and Aquatic Sciences, 38(8), 968–977.

[ece311033-bib-0056] Şahinöz, E. , Doğu, Z. , Aral, F. , Şevik, R. , & Atar, H. H. (2011). Reproductive characteristics of Mullet (*Liza abu* H., 1843) (Pisces Mugilidae) in the Atatürk dam Lake, Southeastern Turkey. Turkish Journal of Fisheries and Aquatic Sciences, 11(1), 7–13.

[ece311033-bib-0057] Santana, F. M. S. (2007). Biologie, Pêche et Dynamique de la Population de Mulet Blanc (*Mugil curema*, Valenciennes, 1836) de Pernambuco‐Brésil. Brest.

[ece311033-bib-0058] Sturm, M. G. D. L. (1978). Aspects of the biology of *Scomberomorus maculatus* (Mitchill) in Trinidad. Journal of Fish Biology, 13(2), 155–172. 10.1111/j.1095-8649.1978.tb03423.x

[ece311033-bib-0059] Tinh, P. H. , MacKenzie, R. A. , Hung, T. D. , Vinh, T. V. , Ha, H. T. , Lam, M. H. , Hanh, N. T. H. , Tung, N. X. , Hai, P. M. , & Huyen, B. T. (2022). Mangrove restoration in Vietnamese Mekong Delta during 2015‐2020: Achievements and challenges. Frontiers in Marine Science, 9, 1043943.

[ece311033-bib-0060] Tran, T. V. T. , Phan, K. L. , & Deivasisamani, B. (2015). The taxonomy key for Mugilidae in Vietnam. Academia Journal of Biology, 37(1), 1–9.

[ece311033-bib-0061] Trisyani, N. (2018). Fishing technique and environmental factors affecting the size of razor clam *Solen* sp. in Indonesia coast. Aquaculture, Aquarium, Conservation & Legislation, 11(1), 29–36.

[ece311033-bib-0062] Unlü, E. , Balcı, K. , & Meriç, N. (2000). Aspects of the biology of *Liza abu* (Mugilidae) in the Tigris River (Turkey). Cybium, 24(1), 27–43.

[ece311033-bib-0063] Vazzoler, A. (1996a). Reproduction biology of teleostean fishes: Theory and practice (p. 169). EDUEM, Brazilian Society of Ichthyology.

[ece311033-bib-0064] Vazzoler, A. E. A. M. (1996b). Biologia da reprodução de peixes teleósteos: Teoria e prática. Eduem.

[ece311033-bib-0065] Veettil, B. K. , Ward, R. D. , Quang, N. X. , Trang, N. T. T. , & Giang, T. H. (2019). Mangroves of Vietnam: Historical development, current state of research and future threats. Estuarine, Coastal and Shelf Science, 218, 212–236.

[ece311033-bib-0066] Wallace, R. A. , & Selman, K. (1981). Cellular and dynamic aspects of oocyte growth in teleosts. American Zoologist, 21(2), 325–343.

[ece311033-bib-0067] Warner, R. R. (1988). Sex change and the size‐advantage model. Trends in Ecology & Evolution, 3(6), 133–136. 21227182 10.1016/0169-5347(88)90176-0

[ece311033-bib-0068] Whitfield, A. (1990). Life‐history styles of fishes in south African estuaries. Environmental Biology of Fishes, 28(1–4), 295–308. 10.1007/BF00751043

[ece311033-bib-0069] Whitfield, A. , Panfili, J. , & Durand, J.‐D. (2012). A global review of the cosmopolitan flathead mullet *Mugil cephalus* Linnaeus 1758 (Teleostei: Mugilidae), with emphasis on the biology, genetics, ecology and fisheries aspects of this apparent species complex. Reviews in Fish Biology and Fisheries, 22(3), 641–681.

[ece311033-bib-0070] Whitfield, A. K. (2015). *Ecological role of Mugilidae in the coastal zone. Biology, ecology and culture of Grey Mullets Mugilidae*, 324.

[ece311033-bib-0071] Wijeyaratne, M. , & Costa, H. (1987). The food, feeding and reproduction of the borneo mullet, *Liza macrolepis* (smith) in a coastal estuary in Sri Lanka. Indian Journal Of Fisheries, 34(3), 283–291.

[ece311033-bib-0072] Wijeyaratne, M. , & Costa, H. (1990). Food and feeding of two species of grey mullets *Valamugil buchanani* (Bleeker) and *Liza vaigiensis* quoy and gaimard inhabiting brackishwater environments in SriLanka. Indian Journal Of Fisheries, 37(3), 211–219.

[ece311033-bib-0073] Wootton, R. J. (1990a). Ecology of teleost fishes. Chapman and Hall.

[ece311033-bib-0074] Wootton, R. J. (1990b). Reproduction. In Ecology of teleost fishes (pp. 159–195). Springer.

[ece311033-bib-0075] Yamamoto, K. (1956). Studies on the formation of fish eggs: I. Annual cycle in the development of ovarian eggs in the flounder, *Liopsetta obscura* . Journal of the Faculty of Science Hokkaido University Series VI Zoology, 12(3), 362–373.

[ece311033-bib-0076] Yamazaki, F. (1965). Endocrinological studies on the reproduction of the female goldfish, *Carassius auratus* L., with special reference to the function of the pituitary gland. Memoirs of the Faculty of Fisheries Hokkaido University, 13(1), 1–64.

[ece311033-bib-0077] Zar, J. H. (1999). Biostatistical analysis. Prentice Hall.

